# The Role of the Adipokine Resistin in the Pathogenesis and Progression of Epithelial Ovarian Cancer

**DOI:** 10.3390/biomedicines10040920

**Published:** 2022-04-16

**Authors:** Klaudia Parafiniuk, Wiktoria Skiba, Anna Pawłowska, Dorota Suszczyk, Aleksandra Maciejczyk, Iwona Wertel

**Affiliations:** 1 Independent Laboratory of Cancer Diagnostics and Immunology, Department of Oncological Gynaecology and Gynaecology, Faculty of Medicine, Medical University of Lublin, Chodźki 4a, 20-093 Lublin, Poland; 50329@student.umlub.pl (K.P.); annapawlowska@umlub.pl (A.P.); dorotasuszczyk@umlub.pl (D.S.); aleksandramaciejczyk@umlub.pl (A.M.); iwona.wertel@umlub.pl (I.W.); 2 Department of Functional Anatomy and Cytobiology, Institute of Biological Sciences, Maria Curie-Sklodowska University, Akademicka 19, 20-033 Lublin, Poland

**Keywords:** obesity, adipokines, resistin, gynecology, immunology, epithelial ovarian cancer

## Abstract

Obesity is a civilization disease associated with an increased risk of developing cardiovascular diseases, diabetes, and some malignancies. The results concerning the relationship between obesity and epithelial ovarian cancer (EOC) are inconclusive. The higher incidence of neoplasms in obese subjects has led to the development of the adipokine hypothesis. Omental adipocyte cells interact with cancer cells, promoting their migration and metastasis via the secretion of adipokines, growth factors, and hormones. One of the adipokines is resistin. It was shown in vitro that resistin stimulates the growth and differentiation of ovarian cancer cells. Moreover, it increases the level of angiogenesis factors, e.g., matrix metalloproteinase 2 (MMP-2) and vascular epithelial growth factor (VEGF). Additionally, resistin induces epithelial–mesenchymal transition (EMT) and stemness in EOC cell lines. A positive correlation has been shown between a higher level of resistin expression and the stage of histological differentiation of EOC or the occurrence of lymph node metastases. In addition, the overexpression of resistin has been found to act as an independent factor determining disease-free survival as well as overall survival in EOC patients. Growing evidence supports the finding that resistin plays an important role in some mechanisms leading to the progression of EOC, though this issue still requires further research.

## 1. Introduction

Epithelial ovarian cancer (EOC) is the most common histological subtype of ovarian cancer and has the highest mortality rate among all gynecological tumors. It constitutes the eighth most common female malignancy [1,2]. EOC is a group of neoplasms with very different pathogenesis and prognosis depending on the histological tumor type and the stage of the disease according to the International Federation of Gynecology and Obstetrics (FIGO) classification [3]. The World Health Organization (WHO) divides EOC into seven categories depending on the cell type: serous carcinomas (SC), mucinous carcinomas (MC), endometrioid carcinomas (EC), clear-cell carcinomas (CCC), transitional-cell Brenner tumors, as well as mixed and undifferentiated types [3]. The serous type can occur as aggressive high-grade serous ovarian carcinoma (HG-SOC), accounting for 90% of cases, or well-differentiated low-grade serous ovarian carcinoma (LG-SOC), accounting for the remaining 10% [4]. The current concept of the WHO classification (the 5th edition) combines histopathology and the immune and molecular profile of ovarian cancer. In the pathogenesis of HG-SOCs and LG-SOCs, there is a described panel of gene mutations, i.e., mutations in the tumor protein p53 (*TP53*), the breast cancer gene 1 and 2 (*BRCA1/2*) genes, homologous recombination deficiency (HRD) mutations for HG-SOCs, and mutations in the neuroblastoma ras viral oncogene homolog (*NRAS*), Kirsten rat sarcoma viral oncogene homolog (*KRAS*), v-raf murine sarcoma viral oncogene homolog B1 (*BRAF*), and human epidermal growth factor receptor 2 (*HER2*) for the latter group [5,6].

According to the Kurman and Shih classification, ovarian tumors can be divided into two clinical types [7]. Type I (25% of cases) includes well-differentiated low-grade serous ovarian carcinomas, mucinous, endometrioid, clear-cell carcinomas, and malignant Brenner tumors. Type II (75% of cases) includes high-grade serous ovarian carcinomas, carcinosarcomas, and undifferentiated carcinomas. Type I has a better prognosis than type II [7,8]. Therefore, the conclusion is that ovarian cancers of types I and II have different pathogenesis, and the latter may even be a metastatic cancer rather than a primary one [8]. The modern approach based on molecular and morphological discoveries indicates that about 80% of HG-SOCs are not primary tumors. In contrast to the traditional assumption, the primary process does not take place in the ovarian surface epithelium or cortical inclusion cysts but rather occurs in the fallopian tube or in the epithelium of the fimbria, to be more specific [5,9,10,11,12,13,14,15].

EOC is diagnosed mostly in advanced stages, with widespread metastasis and poor prognosis, despite therapy [2,16,17]. It has been confirmed that the most important risk factors for EOC include carriage of BRCA1 and BRCA2 gene mutations, hereditary breast and ovarian cancer syndromes, and familial occurrence of hereditary non-polyposis colorectal cancer (Lynch’s syndrome) [18]. The risk of EOC development increases with age and is the highest after menopause, around the seventh decade of life [19]. Moreover, some studies have shown that obesity can promote the development and progression of some types of cancers, including EOC [20,21,22]. A few studies have shown that obesity contributes to the metastasis of ovarian cancer and is negatively correlated with the survival of EOC patients [1,23].

Current obesity data show that more than two billion people worldwide suffer from obesity, which is a factor increasing the risk of the development of cardiovascular diseases, diabetes, and cancer [24,25,26]. Research by the Million Women Study found that obesity is responsible for 5% of all carcinomas in postmenopausal women [27].

Generally, obesity and overweight are defined using the body mass index (BMI). However, the correlation between BMI and obesity-related disease risk is not linear. For example, BMI is only responsible for approximately 17% of the risk of insulin resistance and type 2 diabetes in a population with a BMI of ≥25 [24]. In the light of recent studies, additional factors seem to be much more important in determining the risk of diseases coexisting with obesity, such as epigenetics—especially DNA methylation—socio-economic status, or the natural environment [24].

It has been demonstrated that obesity may be associated with the development of cancer through different pathways. First of all, estrogen produced after menopause mainly by the adipose tissue contributes to the faster proliferation of cancer cells expressing estrogen receptors, especially in breast cancer and cancer of the reproductive organs. Secondly, excess body fat results in excessive levels of insulin-like growth factor and other growth factors, which stimulate the growth and proliferation of cancer cells. The third most important pathway involves the inflammatory process [20].

In obese patients, excess adipose tissue increases the concentration of circulating free fatty acids, adipokines—such as resistin—and many other pro-inflammatory cytokines. The role of resistin in the pathogenesis and progression of EOC consists in modulating the immune system and in influencing cellular signaling pathways. The first mechanism comprises the stimulation of the production of cytokines involved in the pathogenesis of EOC, for instance, tumor necrosis factor α (TNF-α), interleukin 6 (IL-6), and IL-12 [28,29,30]. In the second mechanism, resistin has been found to induce the proliferation of EOC cells through the mammalian target of rapamycin (mTOR), mitogen-activated protein kinase/extracellular signal-regulated kinase (MAPK/ERK), Janus kinase/signal transducer and activator of transcription (JAK/Stat), and phosphatidylinositol 3-kinase/protein kinase B (PI3K/AKT) signaling pathways, triggering an anti-apoptotic effect [31,32]. The above mechanisms contribute to the growth of ovarian cancer cells, increase the invasive potential of these cells, and enhance the secretion of angiogenesis markers, e.g., vascular epithelial growth factor (VEGF) and matrix metalloproteinase 2 (MMP-2) by the tumor cells. They also cause stemness and induce epithelial–mesenchymal transition (EMT) [33]. Chronic low-grade adipose inflammation, which is characteristic of obesity, correlates with increased infiltration of the immune system cells, including T and B lymphocytes, natural killer cells (NK), neutrophils, and monocytes/macrophages (MO/MA).

The majority of macrophages in fat tissue, referred to as adipose tissue macrophages (ATMs), have a pro-inflammatory phenotype (M1). M1 macrophages are known for their antitumor activity, which they owe to the expression of pro-inflammatory and immunostimulatory effector molecules, and the promotion of Th1 response. In contrast, M2 MA exhibit tumor-promoting activity, express a wide range of anti-inflammatory effector molecules, and promote Th2 response as well as angiogenesis and immunotolerance. They also show profibrotic activity [34,35]. M2 macrophages have been shown to promote tumor growth and survival. Consequently, it would seem that they are allies in the fight against cancer. However, local hypoxia in the adipose tissue promotes the conversion of macrophages from M2 to M1 in obese subjects and is believed to be the main maintenance mechanism for chronic inflammation, which in turn triggers a cascade of pro-inflammatory cytokines and carcinogenic adipokines [36]. However, this issue deserves a comprehensive study [37].

Furthermore, it has been shown that a high BMI may increase the likelihood of cancer recurrence, reduce the effectiveness of chemotherapy, increase the toxicity of immunotherapy, reduce its anti-cancer effectiveness, and ultimately increase cancer mortality [20,27,38].

Numerous hypotheses and studies are trying to explain how obesity affects the development and progression of EOC. It should be stressed that data concerning the relationship between EOC and obesity are ambiguous and need further investigation. This paper presents the current state of knowledge of the biological functions and the potential role of resistin, which is produced by the adipose tissue, in the development and progression of ovarian cancer.

The work is a narrative review taking into account the relevant literature in the context of the issue presented in the title. The literature review used the Medline/PubMed and Google Scholar databases and the following keywords: resistin, epithelial ovarian cancer, and obesity.

## 2. The Link between Obesity and EOC

The relationship between obesity and EOC is not well understood yet. In recent years, a few papers have been published that prove the influence of obesity on the development and progression of EOC [39,40]. However, the data on this issue are controversial. Some authors describe an increase in mortality in obese patients, whereas others show an opposite effect or do not indicate a relationship between obesity and ovarian cancer incidence [41,42,43,44].

A meta-analysis conducted by Olsen et al. showed a positive association between obesity and ovarian cancer, and in 10 out of 24 studies, this relationship reached statistical significance. The pooled effect estimate for adult obesity was 1.3 (95%CI1.1“–”1.5), with a smaller increased risk for overweight individuals (OR1.2; 95%CI1.0“–”1.3). The pooled odds ratio (OR) was stronger among case–control studies (OR = 1.5) than in cohort studies (OR = 1.1). There was no evidence that the association varies across different histological types of ovarian cancer [45].

A systematic review and meta-analysis by Hyo Sook Bae et al. showed that obesity in early adulthood (pooled HR 1.67; 95% CI 1.29–2.16) and obesity 5 years before ovarian cancer diagnosis (pooled HR 1.35; 95% CI 1.03–1.76) were associated with poor patient survival. In turn, obesity at the diagnosis of ovarian cancer was associated with worse survival with an increase in BMI only when the BMI was analyzed as a continuous variable (pooled HR 1.02; 95% CI 1.01–1.04) [46].

Other studies have shown that the association between obesity and ovarian cancer is not as strong as for other risk factors, for example, the aberrant expression of the cyclin-dependent kinase 4 inhibitor A (P16INK4a) gene or endometriosis (Table 1) [47]. A few studies have shown a correlation of BMI with the higher risk of development of only certain types of ovarian cancer, including endometrioid, mucinous, clear-cell, and LG-SOC, i.e., tumors belonging to type I according to the Kurman and Shih classification [41,48].

The adipose tissue is increasingly being presented as the largest of the endocrine organs capable of secreting over 50 adipokines, for example, adiponectin, leptin, resistin, visfatin, angiotensinogen, angiopoietin-like protein 2 (ANGPTL2), C-reactive protein (CRP), chemokines, and cytokines, e.g., IL-6 and IL-18, TNF-α, which are involved in metabolic and immune processes [49].

In obese subjects, adipose tissue homeostasis is disturbed. This results in changes in the microenvironment, the profile of inflammatory cells, and secreted proteins. Excessive growth of the adipose tissue changes its histology and function. Some growing adipocytes undergo apoptosis and are surrounded by macrophages. Moreover, there is a change in the M2/M1 macrophage ratio accompanied by adipose inflammation. A shift in the balance of anti-inflammatory macrophages (M2) to pro-inflammatory macrophages (M1) results in increased cytokine production, promoting adipose tissue dysfunction and impaired glucose tolerance. Lipolysis and the secretion of lipids and other substances enhance the inflammatory process [50,51]. Moreover, eosinophils, which infiltrate the adipose tissue in lean subjects, are displaced in the obese ones by neutrophils and mast cells, which in turn augments the release of such inflammatory cytokines as IL-1β, IL-6, and TNF-α, as well as adipokines, including leptin and resistin. It should be stressed that, in humans, resistin is secreted to a small extent by adipocytes, whereas MO/MA with a pro-inflammatory phenotype are its main source [52]. The increase in inflammatory mediators acts as a positive feedback mechanism further recruiting inflammatory cells into the adipose tissue in obese individuals. The entire mechanism leads to chronic low-grade systemic inflammation associated with obesity, called metabolic inflammation. A negative effect of these factors on peripheral tissues is the induction of insulin resistance, hyperinsulinemia, hyperglycemia, hyperlipidemia, vascular damage, and cancer development [53,54]. There are differences in the functioning of the adipose tissue between obese and lean subjects, due to complicated differentiation as obesity develops. It should be emphasized that the secretory state of the adipose tissue may be significantly modified by changes in its cellular composition after weight gain [55,56]. The composition of the fat pad is rearranged, and the phenotypes of pro-inflammatory cells are modulated. These are mainly changes in the number of cells, phenotype, cell localization, and structure. The adipose tissue during these changes is infiltrated by an increasing number of macrophages, which is also related to inflammation of the organism and acquired insulin resistance. Adipokine secretion also differs depending on where the adipose tissue accumulates. This process is characteristic of both humans and animal models [57,58].

It should be emphasized that sustained weight loss reduces the amount of adipose tissue, so the chronic inflammation of obese people is also reduced. The reduction of adipose tissue allows restoring the balance between Th1 and Th2 immunity, which caused the accelerated activation of macrophages in the adipose tissue and the overall pro-inflammatory conditions. More macrophages are stored in visceral adipose tissue, which plays a more important role in insulin resistance. These changes in the adipose tissue microenvironment are not sufficiently understood, and there is no certainty about how T lymphocyte recruitment and macrophage activation work. The lack of available information does not allow us to determine whether adipokine is expressed only by adipose tissue cells or also by inflammatory cells [59,60].

Most differences in adipose tissue functions are connected with cytokines and adipokines secreted by the adipose tissue and with different immune cell populations located within the adipose tissue [58]. These differences are presented in Table 2.

Numerous studies have shown that the adipose tissue plays an important role in the pathogenesis and progression of many cancer types [49,60,61,62,63]. These activities are not always conditioned by the presence of adipose tissue excess in the body. It is worth mentioning that tumor development and metastasis require the interaction of cancer cells with the surrounding tissue. Hence, a tumor should be considered as a tissue phenomenon and not merely as an intracellular disorder. The nature of the adipose tissue causes many tumors to grow, whether directly or indirectly, in contact with the adipose tissue and its components (fibroblasts, connective tissue cells, stem cells, progenitor cells, and numerous signaling elements). The excess of adipose tissue, which promotes obesity, changes the nature of the interactions with cells that differentiate in the neoplasm, significantly changing the cell microenvironment. Many of these changes also resemble those seen in the tumor microenvironment, as the proximity of the adipose tissue can provide a favorable environment for developing tumors, providing a critical link between obesity and carcinogenesis. Another threat is the unique ability of the adipose tissue to expand, which, due to the rapidly growing problem of obesity, is attracting the interest of scientists [52]. EOC is spread by direct infiltration of adjacent organs or by invasion into the pelvic and abdominal cavities. The greater omentum is the most common site of EOC metastasis [1]. At diagnosis, cancer cells are present in the omental tissue in approximately 80% of patients [31,64]. Moreover, cancer cells in this place grow much faster than in the primary tumor. This indicates that omental adipocytes promote the proliferation, migration, and invasion of ovarian cancer cells. This may explain the poor correlation between obesity or overweight and the incidence of EOC. In this case, visceral fat, in particular, near neoplastic cells, may play a key role [31,64].

## 3. Biological Functions of Resistin

Resistin is a cysteine-rich 12-kDa hormone secreted by peripheral blood mononuclear cells (PBMC) and macrophages [65]. There are three names/terms for this protein in the literature: resistin, adipocyte-specific secretory factor (ADSF), and protein found in inflammatory zone 3 (FIZZ3). The name of this adipokine underlines its participation in the pathogenesis of insulin resistance, adipogenesis, and inflammatory processes [66]. It has been well documented that chronic inflammation and an inflammatory milieu play a significant role in EOC progression [67,68,69,70].

The physiological concentration of resistin in the human plasma is 5–20 ng/mL, but it increases with age and changes in medical conditions, e.g., in diabetic patients, it is approx. 40 ng/mL [71,72]. In obese subjects, when obesity is the result of both a high-calorie diet and genetic disorders, resistin concentration is higher than in lean individuals. The rate of visceral adiposity is significantly correlated with the serum levels of resistin and other adipokines [73]. Moreover, hyperglycemia also increases plasma resistin concentrations [74]. Resistin circulates in trimeric and oligomeric forms. Oligomeric resistin has a greater effect on the induction of inflammation [65]. Its serum concentration increases not only in obese subjects but also during chronic inflammation [75]. Several single-nucleotide polymorphisms (SNPs) in the human resistin gene (*RETN*) also exert an effect on its plasma concentration [65]. Resistin synthesis is probably induced by various inflammatory stimuli such as lipopolysaccharide (LPS), IL-6, IL-1β, TNF-α, and resistin itself [76]. There are only two receptors for human resistin, i.e., toll-like receptor 4 (TLR4) and adenylyl cyclase-associated protein 1 (CAP1) [77]. The binding of resistin to CAP1 increases the expression of NF-κB, cAMP, and protein kinase A, enhancing the inflammatory response involved in carcinogenesis. These signaling pathways lead to an increase in the transcription of pro-inflammatory cytokine genes for IL-1, IL-6, and TNF-α involved in carcinogenesis. These cytokines enhance cell proliferation and stimulate angiogenesis and metastasis [78]. Resistin activates intracellular signaling pathways such as PI3K/Akt/mTOR and Ras/Raf/MEK/ERK, leading to the inhibition of apoptosis. It was shown that resistin reduces the expression of the proapototic genes *FADD*, *FAS*, and *CASP8* in ovarian cells which promote the growth of cancer. This adipokine also increases the metastatic potential of EOC by inhibiting tumor suppressor miRNAs (miR), such as miR-200c and miR-186 [79,80]. Therefore, increasing evidence links human resistin to the low-grade chronic subclinical inflammation and high levels of macrophage infiltration observed in the adipose tissue of obese individuals, rather than to the fatty deposits themselves [81]. It is, therefore, postulated that resistin mediates the recruitment of other immune cells by stimulating the action of pro-inflammatory mediators. The immunological processes in this case are, therefore, self-propelling [81,82].

It has been documented that resistin increases the activity of some pathways of the immune system and weakens others. Resistin attenuates the effects of dendritic cells (DCs) and T lymphocytes by affecting regulatory T cells (Tregs). Treg lymphocytes, which express CD4 and CD25, change the tumor environment by secreting cytokines such as tumor growth factor β (TGF-β) and IL-10 [83]. In healthy people, the mechanisms responsible for suppressing the immune system’s response to antigens in the tumor microenvironment inhibit the response to tumor antigens and contribute to the escape of a tumor from immune surveillance. Activation of Tregs is triggered by the chemokine (C–C motif) ligand 28 (CCL28), the concentration of which increases in states of tissue hypoxia [84,85]. Consequently, tumor growth by inducing tissue hypoxia fuels the vicious cycle of immunogenicity decline and tumor growth. Another chemokine involved in the activation of Tregs is chemokine (C–C motif) ligand 22 (CCL22). A high concentration of CCL22 has been demonstrated in the peritoneal fluid of patients with ascites in the course of ovarian cancer [85,86].

This may be the cause of a weaker immune response to the antigens of the developing neoplasm [87]. Moreover, resistin stimulates an inflammatory response in MO/MA, antagonizes the anti-inflammatory effect of adipokine, and increases the production of pro-inflammatory IL-12 and TNF-α. These mechanisms have been identified as important in the development of atherosclerosis [88]. Resistin can also regulate the cell cycle by holding the progression of cells from one phase of the cycle to the next. Indirectly, through activation of ERK, it leads to dysregulation of the suppressor of cytokine signaling 3 (SOCS3) and then to JAK2/STAT3 pathway downregulation. Ultimately, cells are arrested in the G1 phase [89].

The activities of resistin contribute to the emergence of diseases such as obesity, type 2 diabetes mellitus, atherosclerosis, and some types of neoplasms. Several studies have shown a higher resistin level in some cancer types, including esophageal squamous cancer, malignant lymphoma, gastric, colorectal, breast, and endometrial cancer [79,90,91]. Furthermore, resistin can promote the proliferation of prostate cancer cells [92]. It has also been proven that resistin is an independent prognostic factor in pancreatic adenocarcinoma. Recent research has shown the effect of resistin on worsening the prognosis in patients with lung adenocarcinoma [92,93]. Moreover, a positive correlation between the expression of resistin in neoplastic tissue and the histological differentiation (grade) of a tumor, the tumor size, and the condition of the lymph nodes has been demonstrated [92]. A similar correlation has been shown between the stage of a disease and the concentration of resistin in the serum [94]. No correlation has been found between the level of resistin expression and the age of the patients at diagnosis, their smoking and drinking habits, and their blood type [92]. However, some reports linked resistin to some types of gender-related cancer, which mainly occur in women, e.g., breast cancer and endometrial cancer [62,95]. In turn, no significant gender-related differences in the resistin level were reported in the case of some cancer types that mainly affect males. This may indicate a special role of resistin in the pathogenesis of gynecological neoplasms. It is worth mentioning that the level of resistin in the tumor microenvironment of neoplastic tissues was higher than in the serum and was correlated with tumor progression [63]. It has been shown in vitro that resistin stimulated the growth and differentiation of new cell colonies using ovarian cancer cell lines. Moreover, it induced resistance to cisplatin and increased the level of angiogenesis markers such as MMP-2 and VEGF [33]. According to the available literature, resistin acts as an angiogenesis-stimulating factor by regulating several pathways:

- the PI3K–Akt signaling pathway in chondrosarcoma cells, through downregulation of microRNA expression (miR)–16–5p [96];
- the PI3K–Akt–Sp1 pathway in ovarian cancer cells (HO-8910) through increasing the interaction with Specificity protein 1 (Sp1), triggering progressive phosphorylation of Sp1 on Thr453 and Thr739 [97];
- ERK, c-Jun N-terminal kinase (JNK), and p38 pathways activation in osteocarcinoma cells [98];
- TLR4, p38, MAPK, and transcription factor nuclear factor-kappa B (NFκβ) in gastric cancer cells through induction of the expression of stromal cell-derived factor 1 (SDF1)/C–X–C motif chemokine 12 (CXCL12) [78,99].

The first three pathways induce angiogenesis through a VEGF-A-dependent mechanism. The increased expression of this factor results in increased angiogenesis [96,97,98]. The last pathway does so through a C–X–C chemokine receptor type 4 (CXCR4)-dependent mechanism and by recruiting endothelial progenitor cells (EPCs) from the bone marrow [78,99].

**Figure 1:**
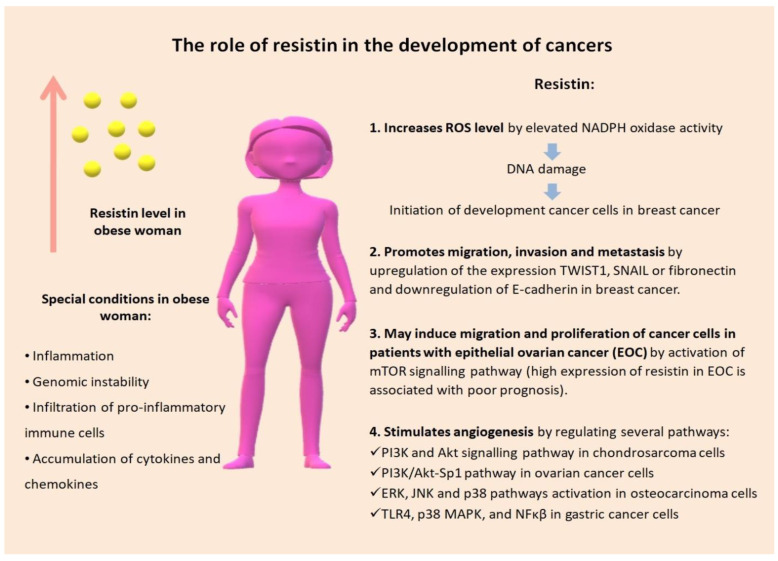
The complex role of resistin in cancer development [31,78,96,97,98,99,100]. Reactive oxygen species (ROS); nicotinamide adenine dinucleotide phosphate (NADPH); mammalian target of rapamycin (mTOR); phosphatidylinositol 3-kinase (PI3K); protein kinase B (Akt); specificity protein 1 (Sp1); extracellular signal-regulated kinase (ERK); c-Jun N-terminal kinase (JNK); toll-like receptor 4 (TLR4); mitogen activated protein kinase (MAPK); transcription factor nuclear factor-kappa B (NFκβ).

Resistin has been investigated also in terms of its proliferation-stimulating activities. Signaling pathways connecting resistin with cancer include those related to TLR4, PI-3K, and NFκβ [90]. In several cancer types, e.g., in prostate cancer, lung cancer, gastric cancer, melanoma, and ovarian cancer, the activation by resistin of diverse signaling pathways has been shown, such as those associated with PI-3 K, NFκβ, epidermal growth factor receptor (EGFR), TLR4 receptor, IL-6-dependent STAT3 signaling, pAKT, and Caveolin 1 (Cav-1); it was also revealed also that the presence of miR let-7a, miR-200c, and miR-186 was associated with proliferation [90]. Additionally, resistin induced EMT and stemness in the ovarian cancer cell lines A2780 and SKOV3 [92]. The complex role of resistin in cancer development is presented in Figure 1.

## 4. The Role and Significance of Resistin in the Biology and Progression of Epithelial Ovarian Cancer

It should be stressed that there are only a few indications in the literature that associate resistin with the progression of ovarian cancer [20,33,97]. In a study by Li Pang and Xiaohan Chang on 50 EOC specimens, it was shown that higher resistin expression is positively correlated with the histological differentiation of the tumor and the occurrence of lymph node metastases [31]. No similar relationship was found between the level of resistin and the histological type of the tumor, the FIGO stage, age, the residual tumor after initial laparotomy, and the level of the antigen Ca125 in the serum [31]. Moreover, it was proven that the level of resistin expression is an independent factor determining the disease-free survival as well as the overall survival of EOC patients. In addition, it was shown that administration of exogenous resistin induced the proliferation of SKOV3 and CAOV3 ovarian cancer cells, while the administration of exogenous rapamycin, which is an inhibitor of resistin, inhibited their proliferation. In the above-mentioned study, resistin increased the proliferation of SKOV3 and CAOV3 cells through the mTOR signaling pathway. The experiment also proved that resistin promotes the migration of ovarian cancer cells [31].

Resistin disturbs the homeostasis of the body through various mechanisms and can promote the progression of EOC by both modulating the immune system and affecting cellular signaling pathways. It stimulates the production of TNF-α and interleukins IL-6 and IL-12 in adipocytes as well as in immune system cells involved in the pathogenesis of ovarian cancer [28,29,30]. In addition, ovarian tumor cells also produce these factors. It was shown in vitro that the levels of TNF-α and IL-6 mRNA were 1000-fold higher in ovarian cancer cells than in normal ovarian epithelial cells [101]. Results showed a higher concentration of tumor necrosis factor receptor 2 (TNFR2) in ovarian cancer cells than in normal cells [102]. In turn, IL-12 decreases the synthesis of angiostatic factors [30]. In addition to a pathogenetic effect, these cytokines influence the occurrence of paraneoplastic syndromes. IL-6 causes fever, hypercalcemia, cachexia, and thrombotic syndromes. TNF-α causes fever, cachexia, and anemia [30]. Elevated levels of these cytokines increase the activity of the Notch signaling pathway, which is known to induce angiogenesis in the ovary and contributes to the progression of EOC [103]. Interestingly, the targeting of anti-IL-6 and anti-TNF-α antibodies to EOC cells in culture led to a reduction in neovascularization and tumor growth [104].

Other investigations have revealed that resistin stimulates the growth of ovarian cancer cells, increases their invasive potential, and enhances the secretion of the angiogenesis markers VEGF and MMP-2 by tumor cells. It also causes stemness and induces EMT and resistance to cisplatin [33]. The most common treatment for ovarian cancer patients is cisplatin-based chemotherapy. The development of chemoresistance is a major contributing factor in treatment failure and metastases. Qiu et al. reported that resistin may play an important role in the development of cisplatin resistance and invasiveness in ovarian cancer cells by the induction of EMT and stemness. The underlying mechanisms are not fully understood, but resistin may be responsible for inducing stemness markers like sox2, oct4, and nano or suppressing miR such as miR-186, miR-200c, and miRNAs let-7. Moreover, resistin can promote EMT and stemness by the upregulation of zinc finger E-box binding homeobox 1 (ZEB1) or vimentin and also by the downregulation of E-cadherin [33]. In addition, while assessing the effect of resistin on porcine ovarian cells, Rak et al. [32] found that it had no effect on cell proliferation, reduced proapoptotic gene expression, inhibited caspase 3 activity and DNA fragmentation, and activated the MAPK/ERK, JAK/Stat, and Akt/PI3K signaling pathways, thus producing an anti-apoptotic effect [32]. Additionally, resistin stimulates the production of monocyte chemoattractant protein-1 (MCP-1), endothelin-1, and MMPs, as well as stimulates the expression of adhesion molecules, such as vascular cell adhesion molecule 1 (VCAM1), intercellular adhesion molecule 1 (ICAM-1), and pentraxin 3, which participate in leukocyte adhesion [28]. These proteins are involved in the process of both regional invasion and distant metastasis. Resistin also stimulates the proliferation and migration of endothelial cells and vascular smooth muscle cells (VSMC) [65]. A study conducted by Santilli et al. [105] showed that resistin promotes insulin resistance by blocking the peroxisome proliferator-activated receptor γ (PPAR-γ) and increases oxidative stress, endothelial dysfunction, and the activation of platelets [105]. PPAR-γ is also found in the ovary, where it plays an important role in the proper function of the female gonad. Inhibition of PPAR-γ expression leads to infertility [106]. This mechanism may also be affected by ovarian carcinogenesis [107]. PPAR-γ in the ovary is involved in the regulation of angiogenesis, inflammation, and the cell cycle. PPAR-γ agonists, i.e., ciglitazone and troglitazone, have been shown to inhibit the proliferation of ovarian cancer cell lines by arresting the cell cycle. Another PPAR-γ agonist, pioglitazone, has been found to induce apoptosis of ovarian tumor cells [107]. The evaluation of intracellular signaling pathways in ovarian cancer cells showed elevated activity of PI3/Akt, mTOR, and AMPK kinases [107]. The PI3/Akt kinase pathway is affected by resistin, which enhances VEGF expression and angiogenesis. Therefore, a thesis has been put forward that anti-angiogenic therapy may be a beneficial treatment against ovarian tumors, especially in obese patients [97].

## 5. Conclusions

Taking into account the rising global obesity epidemic, it is a necessity to gain insight into the immunomodulatory effects of obesity within the ovarian cancer microenvironment. We hypothesize that obesity-associated inflammation can modulate the immune response in EOC patients and may affect the results of immunotherapy. Therefore, it is critical to find differences in the TME of obese and lean patients. The current literature data still provide limited evidence directly linking the effect of resistin to the development of ovarian cancer. The effects of resistin on adipocytes and immune cells and its role in inducing low-intensity chronic inflammation are much better studied.

Given the complex nature and heterogeneity of the inflammatory milieu of EOC and inconsistent evidence of the relationship between resistin, obesity, and ovarian cancer risk, further studies should be carried out. Perhaps in the future, resistin will become a prognostic, a predictor, or a target of therapy in obese patients with ovarian cancer.

## Figures and Tables

**Table 1:** Factors influencing the risk of developing EOC (OR + 95% CI) [47].

Variables	Odds Ratio (95 % CI)
P16INK4a	2.657 (1.173–6.014)
Polycystic ovarian syndrome	1.580 (1.081–2.310)
Endometriosis	1.433 (1.294–1.586)
**Obesity**	**1.274 (1.194–1.36)**
Hormone therapy (estrogen-progestin)	1.190 (1.043–1.357)
**Overweight**	**1.079 (1.041–1.119)**
BRCA2 N372H rs144848	1.079 (1.018–1.143)
MTHFR C677T	1.077 (1.032–1.124)
Recreational physical activity	0.830 (0.745–0.925)
Oral contraceptive	0.655 (0.515–0.833)
Breast feeding	0.719 (0.679–0.762)

Odds ratio (OR); cyclin-dependent kinase 4 inhibitor A (P16INK4a); breast cancer gene 2 (BRCA 2); methylenetetr hydrofolate reductase (MTHFR).

**Table 2:** Differences in the functioning of the adipose tissue in obese and lean subjects [58].

Differences in the Functioning of Adipose Tissue	Lean	Obese
Immune Cell Populations	ILC2s, Tregs, eosinophils, type II NKT and Th2 cells, and M2-like macrophages	Th1, NK, CD8^+^ T cells, adipocyte MHC II, M1-like macrophages
Cytokines	IL-4, IL-5, IL-10, IL-13, IL-25, IL-33	IL-1β, IL-6, TNFα, IFN-γ
Adipokines	Adiponectin, Sfrp5	Leptin

Group 2 innate lymphoid cells (ILC2s); regulatory T cells (Tregs); T helper cells (Th); interleuikn (IL); natural killer T cells (NKT); secreted frizzled-related protein 5 (Sfrp5); natural killer cells (NK); major histocompatibility complex (MHC); tumor necrosis factor α (TNFα); interferon-γ (IFN-γ).

## Data Availability

All data generated or analyzed during this study are included in this publication.
